# Identification of three ADA2 deficiency families with novel *CECR1* mutations

**DOI:** 10.1186/1546-0096-13-S1-P153

**Published:** 2015-09-28

**Authors:** G Sarrabay, A Insalaco, F Uettwiller, N Tieulié, P Quartier-dit-maire, J Melki, I Touitou

**Affiliations:** 1 CHU Montpellier, Montpellier, France

## Introduction

Adenosine desaminase 2 deficiency (DADA2) is a rare monogenic autoinflammatory disease, presenting with systemic inflammation, vascular pathology with possible strokes, and mild immunodeficiency. Recently, the *CECR1* gene was found to be responsible for this disease with an autosomal recessive inheritance.

## Objective

We report new mutations identified in 3 families referred to our laboratories for genetic diagnosis.

## Patients and methods

In family 1 (French), the proband, a 9 year-old girl, exhibited fever, pseudo periarteritis nodosa (PAN) and several strokes. Her 7-year old sister presented with fever, anemia and neurological impairments (headaches, drowsiness and third cranial nerve palsy). In family 2 (Italian), the index-case had recurrent fever, arthromyalgia, and livedo reticularis of trunk and limbs. In family 3 (Tunisian), consanguineous brother and sister exhibited pleiomorphic vascularitis features (PAN, livedo, renal microaneurysms) with inflammation and anemia. Parents in all families were asymptomatic.

Using double-strand Sanger sequencing (ABI3130x, Life Technologies), we searched for point mutations in the *CECR1* coding regions. Quantitative polymerase chain reaction (qPCR) analysis was subsequently performed (LightCycler, Roche) when only one point mutation was identified (family 1).

## Results

The two sisters of family 1 harbored two compound heterozygous mutations: p.Tyr453Cys (published) and deletion of the entire exon 7 (novel). We identified a new variant in family 2: p.Arg49Glyfs*4 (c.144del), and p.Thr360Ala, a published mutation. In family 3, we found a homozygous substitution, p.Gly25Cys in both siblings. This new variant was predicted to be benign by different algorithms but was absent from general databases (allele frequency <10^-4^ in ExAC). Exome analysis was performed and this variant was the only one to be retained after the various filters. However, this variant remains controversial until extensive family investigation is performed. We confirmed that each parental allele carried a mutation in the 3 families.

## Conclusion

We report here 3 new variants in the *CECR1* gene. Since DADA2 is an auto-inflammatory disease with original neurological involvement, *CECR1* testing should be widely performed in patients with such a phenotype. Both point mutations and deletions have to be searched.

## Consent to publish

Written informated consent for publication of their clinical details was obtained from the patient/parent/guardian/relative of the patient.

